# Use of mechanical lithotripter to assist in the endoscopic removal of an impacted long chicken bone in sigmoid diverticula

**DOI:** 10.1055/a-2106-0612

**Published:** 2023-07-11

**Authors:** Eduardo Rodrigues-Pinto, Emanuel Dias, Renato Medas, Guilherme Macedo

**Affiliations:** 1 Gastroenterology Department, Centro Hospitalar Universitário de São João, Porto, Portugal; 2 Faculty of Medicine of the University of Porto, Porto, Portugal

A 56-year-old woman without relevant medical history was admitted to the emergency room with persistent lower left abdominal pain for the previous 15 days. Abdominal computed tomography revealed diverticulosis of the left colon, with a 55-mm linear foreign body in the sigmoid colon, located transversely to the colon axis, with thickening of the bowel wall and adjacent fat, without perforation ( Fig. 1 ). The patient was referred for endoscopic removal.

**Fig. 1:**
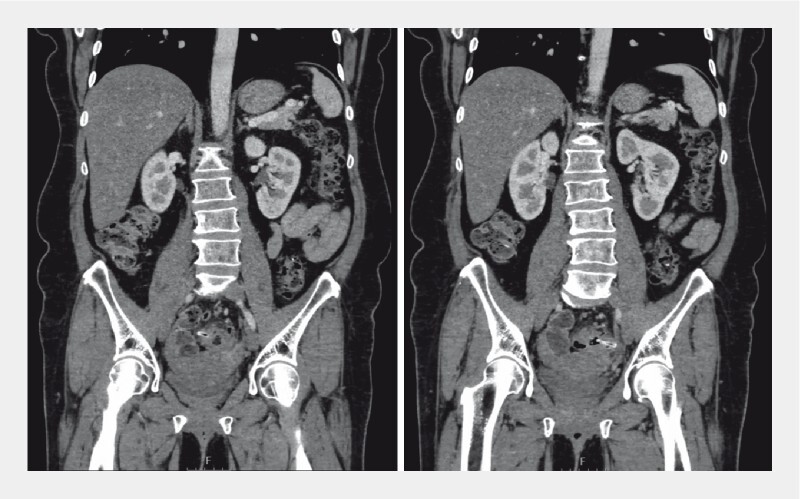
Coronal abdominal computed tomography showing the chicken bone measuring 55 mm in length located in the sigmoid colon, transversely oriented to the colon axis, without signs of perforation.

The impacted bone was stuck crosswise, 30 cm from the anal verge, with both ends embedded in diverticula on opposite walls ( Fig. 2 ). Multiple removal attempts with a foreign body forceps were unsuccessful. We decided to use a mechanical lithotripter to assist in endoscopic removal ( Video 1 ).

**Fig. 2:**
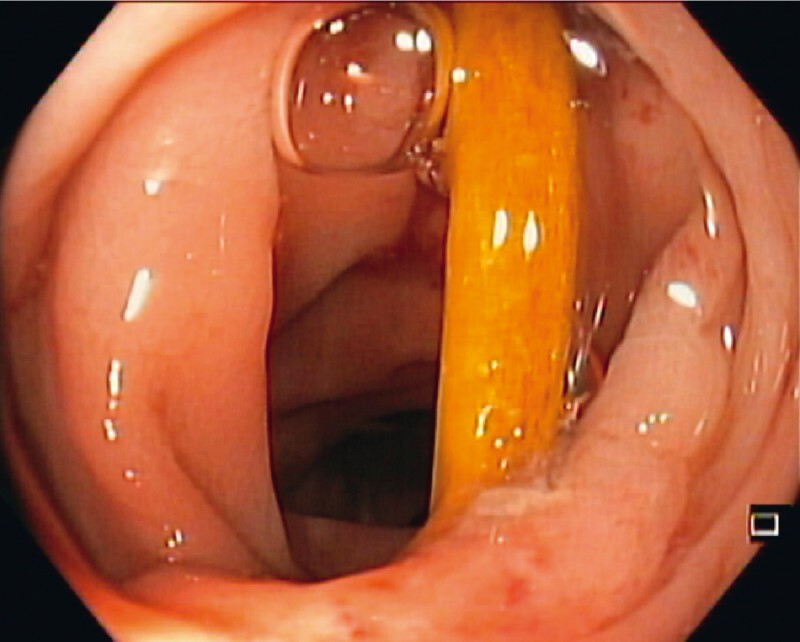
Endoscopic image of the chicken bone located crosswise, 30 cm from the anal verge, with both ends embedded in diverticula on opposite walls.

**Video 1** Use of mechanical lithotripter to assist in the endoscopic removal of an impacted long chicken bone in sigmoid diverticula.


A 0.035-inch guidewire was looped around the bone by advancing the guidewire on one side of the bone and capturing the distal tip on the other side. With the two ends of the guidewire outside the anus, a lithotripter cable was advanced over the ends until the tip reached the impacted bone. Lithotripter positioning on the center of the bone was difficult due to angulation of the colon, with constant catching of the mucosa near one end of the bone ( Fig. 3 ). A foreign body forceps was used to grab the guidewire and position it centrally while adjusting the lithotripter cable ( Fig. 4 ). The lithotripter handle was then attached to the cable and progressively ratcheted down until the guidewire cut through the bone. The bone could then be easily removed with foreign body forceps ( Fig. 5 ). Mucosal ulceration was seen, without contrast extravasation on fluoroscopy. The patient was discharged home the following day without symptoms and remains well 4 months later.

**Fig. 3:**
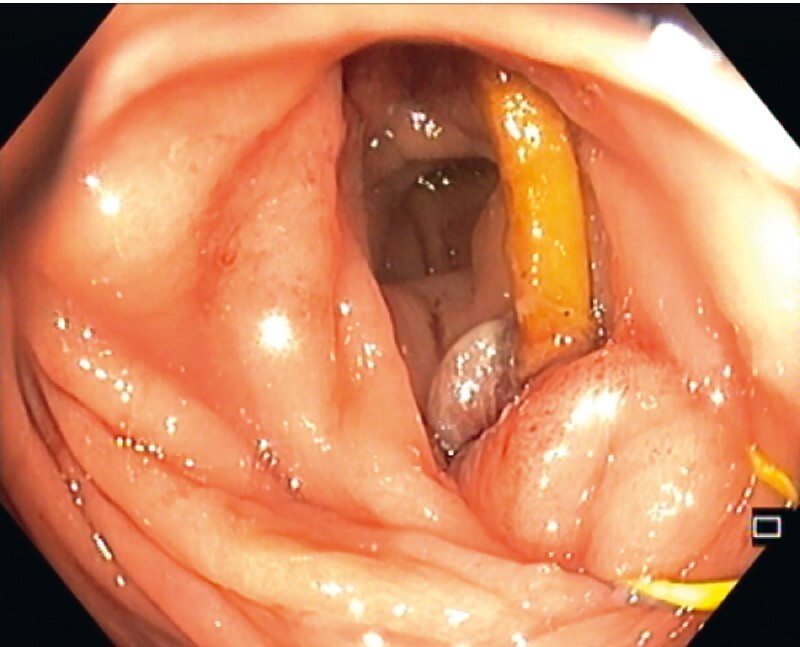
Constant catching of the mucosa near one end of the bone while positioning the lithotripter, despite several changes in scope and patient position.

**Fig. 4:**
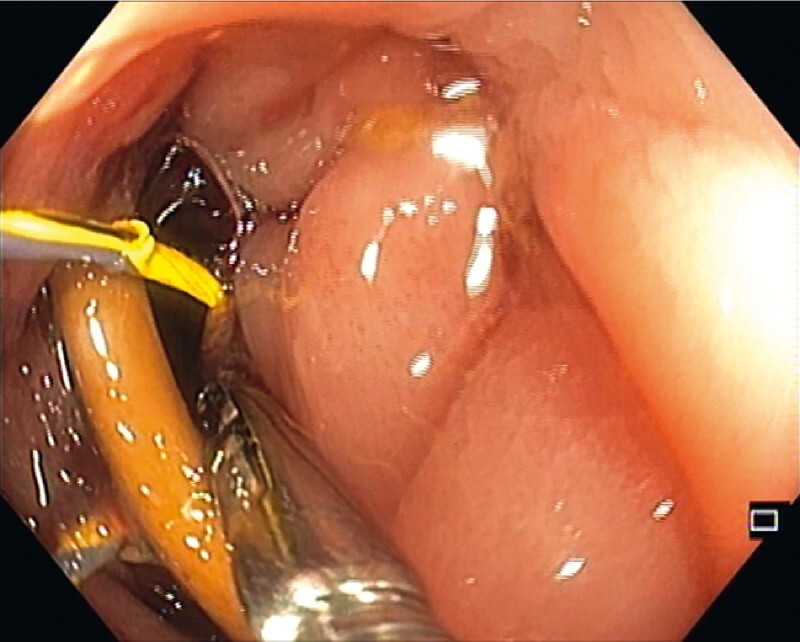
Mobilization of the looped guidewire with a foreign body forceps onto the center of the bone, while adjusting the lithotripter cable, to ensure closure without mucosal entrapment.

**Fig. 5:**
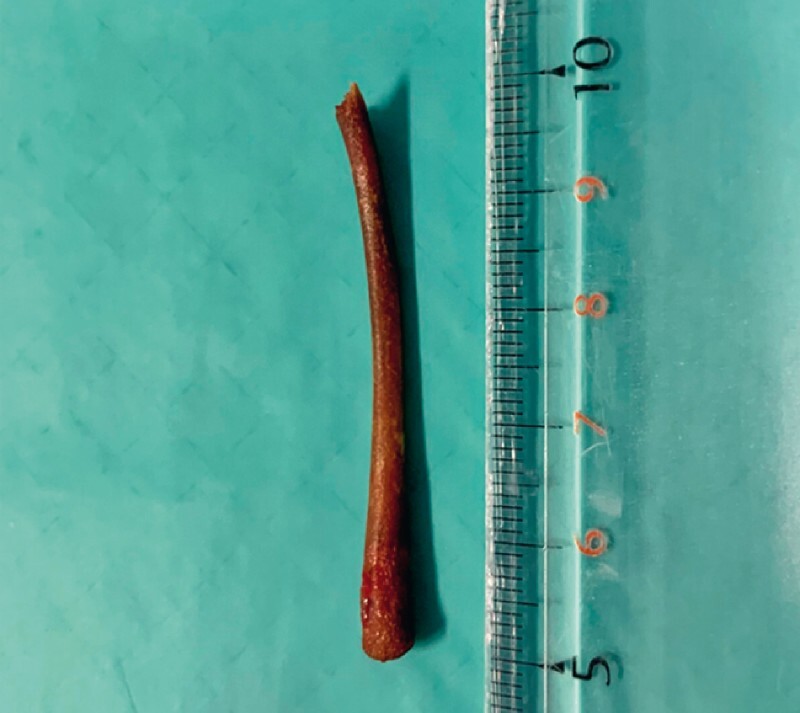
The retrieved foreign body after cutting one end of the bone with the mechanical lithotripter.

Most foreign bodies can pass through the gastrointestinal tract without consequence, but obstruction can occur 1 . The mechanical lithotripter-assisted technique 2 allowed us to cut the bone, facilitating subsequent removal without causing complications.

Endoscopy_UCTN_Code_TTT_1AQ_2AH

## Notes

Endoscopy E-Videos

https://eref.thieme.de/e-videos
*E-Videos* is an open access online section of the journal *Endoscopy* , reporting on interesting cases and new techniques in gastroenterological endoscopy. All papers include a high-quality video and are published with a Creative Commons CC-BY license. Endoscopy E-Videos qualify for HINARI discounts and waivers and eligibility is automatically checked during the submission process. We grant 100% waivers to articles whose corresponding authors are based in Group A countries and 50% waivers to those who are based in Group B countries as classified by Research4Life (see: https://www.research4life.org/access/eligibility/ ). This section has its own submission website at https://mc.manuscriptcentral.com/e-videos

## References

[1] Church J How to remove an impacted chicken bone from the sigmoid colon endoscopically Dis Colon Rectum 2000 43 1018 1019 10910254 10.1007/BF02237371

[2] Kedia P Jacob B DiMaio C J Mechanical lithotriptor-assisted endoscopic removal of an eroded gastric lap band Gastrointest Endosc 2015 81 1256 1257 25442081 10.1016/j.gie.2014.08.018

